# Rumen Methanogenesis, Rumen Fermentation, and Microbial Community Response to Nitroethane, 2-Nitroethanol, and 2-Nitro-1-Propanol: An In Vitro Study

**DOI:** 10.3390/ani10030479

**Published:** 2020-03-13

**Authors:** Zhenwei Zhang, Yanlu Wang, Xuemeng Si, Zhijun Cao, Shengli Li, Hongjian Yang

**Affiliations:** State Key Laboratory of Animal Nutrition, College of Animal Science and Technology, China Agricultural University, Beijing 100193, China; qingyibushuo@163.com (Z.Z.); yanluwang@yeah.net (Y.W.); sxmswun@126.com (X.S.); caozhijun@cau.edu.cn (Z.C.); lisheng0677@163.com (S.L.)

**Keywords:** nitrocompounds, methanogenesis, rumen fermentation, microbial community, coenzyme

## Abstract

**Simple Summary:**

The present study comparatively investigates the inhibitory difference of nitroethane (NE), 2-nitroethanol (NEOH), and 2-nitro-1-propanol (NPOH) on in vitro rumen fermentation, microbial populations, and coenzyme activities associated with methanogenesis. The results showed that both NE and NEOH were more effective in reducing ruminal methane (CH_4_) production than NPOH. This work provides evidence that NE, NEOH, and NPOH were able to inhibit methanogen population and dramatically decrease methyl-coenzyme M reductase gene expression and the content of coenzymes F_420_ and F_430_ with different magnitudes in order to reduce ruminal CH_4_ production.

**Abstract:**

Nitroethane (NE), 2-nitroethanol (NEOH), and 2-nitro-1-propanol (NPOH) were comparatively examined to determine their inhibitory actions on rumen fermentation and methanogenesis in vitro. Fermentation characteristics, CH_4_ and total gas production, and coenzyme contents were determined at 6, 12, 24, 48, and 72 h incubation time, and the populations of ruminal microbiota were analyzed by real-time PCR at 72 h incubation time. The addition of NE, NEOH, and NPOH slowed down in vitro rumen fermentation and reduced the proportion of molar CH_4_ by 96.7%, 96.7%, and 41.7%, respectively (*p* < 0.01). The content of coenzymes F_420_ and F_430_ and the relative expression of the *mcr*A gene declined with the supplementation of NE, NEOH, and NPOH in comparison with the control (*p* < 0.01). The addition of NE, NEOH, and NPOH decreased total volatile fatty acids (VFAs) and acetate (*p* < 0.05), but had no effect on propionate concentration (*p* > 0.05). Real-time PCR results showed that the relative abundance of total methanogens, *Methanobacteriales*, *Methanococcales,* and *Fibrobacter succinogenes* were reduced by NE, NEOH, and NPOH (*p* < 0.05). In addition, the nitro-degradation rates in culture fluids were ranked as NEOH (−0.088) > NE (−0.069) > NPOH (−0.054). In brief, the results firstly provided evidence that NE, NEOH, and NPOH were able to decrease methanogen abundance and dramatically decrease *mcr*A gene expression and coenzyme F_420_ and F_430_ contents with different magnitudes to reduce ruminal CH_4_ production.

## 1. Introduction

Nitrocompounds are classified into aromatic compounds containing nitro groups in the aromatic ring and aliphatic–aromatic compounds containing nitro groups only in the aliphatic side chain. Among the naturally occurring aliphatic nitrocompounds, 3-nitro-1-propanol (3-NPOH) and 3-nitro-1-propionic acid (NPA) are regarded as nitrotoxins that can accumulate to toxic levels in certain forages (e.g., *Astragalus*); their toxic and metabolic properties were well investigated in ruminants and monogastric animals nearly a century ago [1]. Over the last decades, certain commercially available short-chain aliphatic nitrocompounds have been chemically synthesized and are known to be potent methane-inhibiting compounds, since methane (CH_4_) from livestock is increasingly considered as a significant greenhouse gas [2]. 

Among these commercially available nitrocompounds, Latham et al. [2] summarized the supplemental effects of nitroethane (NE), 2-nitroethanol (NEOH), and 2-nitro-1-propanol (NPOH) in comparison with 3-NPOH and NPA on CH_4_ and volatile fatty acid (VFAs) production during in vitro rumen incubations, finding that the yield of VFAs was almost not affected, whereas CH_4_ production consistently decreased by up to 58%–99%. Regarding in vivo studies in sheep [3] and feedlot steer [4,5,6], CH_4_-reducing activity varied depending on dose levels and depletion of nitrocompounds. More recently, in the study of Hristov et al. [7], as much as a 64-fold increase in hydrogen (H_2_) production was observed in 3-nitrooxypropanol-treated cattle, which was still only about 3% of the H_2_ spared from ruminal production. Despite the fact that CH_4_ emission is negatively associated with energy retention and greenhouse gas production, rumen archaea play an important ecological role in methanogenesis, however, few studies have examined the microbial response to nitrocompounds. In many of the aforementioned studies, the inhibited archaea populations or methanogenesis-associated enzymes were not characterized though methyl-coenzyme M reductase (*mcr*). The coenzymes F_420_ and F_430_ are known in nature as key enzymes involved in CH_4_ formation from H_2_ and CO_2_ by archaea [8,9,10]. Although the naturally occurring nitrocompounds 3-NPOH and NPA are recognized to be metabolized by rumen microorganisms to aminopropanol and *β*-alanine, a nonessential amino acid may be utilized by ruminant animals and potentially used as a source of carbon, nitrogen, and energy, making it an attractive candidate [1,2]. To avoid unknown risks of toxic intermediates in feeding practice trials regarding commercially available NE, NEOH, and NPOH, the present study comparatively investigated the inhibitory difference of each nitrocompound on in vitro rumen fermentation, microbial populations, and coenzyme activities associated with methanogenesis.

## 2. Materials and Methods

The donor animals and experimental procedures were approved by the requirements of Beijing Municipal Council on Animal Care according to the protocol of CAU20171014-1.

### 2.1. Nitrocompounds

**Figure 1:**
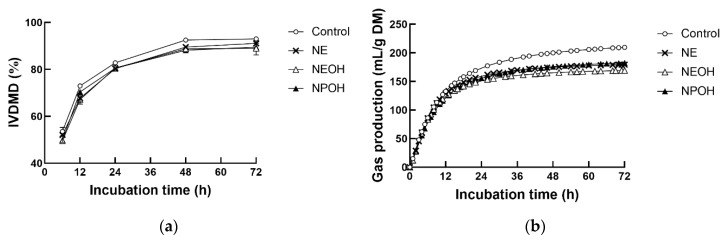
Effect of NE, NEOH, and NPOH addition to culture fluids on in vitro dry matter disappearance (IVDMD (**a**) and cumulative gas production (**b**) of grain-rich feed incubated with rumen fluids obtained from lactating dairy cows. NE: nitroethane; NEOH: 2-nitroalcohol; NPOH: 2-nitro-1-propanol. Statistical analyses showed that the effects of nitrocompounds on IVDMD and gas production were significant at *p* < 0.01.

Nitroethane (NE), 2-nitroethanol (NEOH), and 2-nitro-1-propanol (NPOH) were purchased commercially (Sigma Aldrich, Inc., LA, USA). Structures of these nitrocompounds are shown in Figure 1. Their analytical grades were 99%, 90%, and 98%, respectively.

### 2.2. Animals

Five multiparous and rumen-cannulated Holstein lactating dairy cows (540 ± 25.3 kg body weight; 100 ± 8.5 days in milk, 33.0 ± 0.78 kg/d milk yield; mean ± SD) were selected as the donors of rumen fluid. Cows were maintained on a total mixed ration (calculated as % dry matter basis) of alfalfa hay (16.32%), whole corn silage (24.61%), 1 kg corn meal (3.95%), extruded corn (19.60%), soybean meal (14.38%), soybean hull (4.09%), extruded soybean (3.43%), whole cottonseed (8.25%), trace mineral, and vitamin premix (5.37%), according to the Chinese Feeding Standard of Dairy Cow (NY/T 34 2004). 

### 2.3. In Vitro Experiment

In vitro fermentations in anaerobic glass bottles (volume capacity of 120 mL) incubated with rumen fluids were performed following the previous description of Zhang and Yang [11]. The treatments included the control (no additive treatment), 10 mmol/L of NE, 10 mmol/L of NEOH, and 10 mmol/L of NPOH. Corn meal and alfalfa hay (500 mg; 80:20, w/w) were used as the fermentation substrates. 

Rumen fluids were collected before morning feeding from each rumen-cannulated donor cow into a pre-warmed thermos flask at 39 °C. After filtering through 4 layers of cheesecloth and mixed in equal proportion, 25 mL of rumen fluids were incubated into anaerobic glass bottles with 50 mL buffered medium (pH 6.8) [12]. The batch cultures were performed at 39 °C in both automated and manual systems. In the automated system, five bottles per treatment were connected to the gas inlets of an automated gas recording system (AGRS) and continuously incubated for 72 h to continuously record cumulative gas production (GP). In the manual system, five bottles per treatment were connected to pre-emptied air bags to collect fermentation gas samples and removed at 6, 12, 24, 48, and 72 h of incubation. The batch cultures were repeated and completed in three consecutive runs. One milliliter of gas sample was drawn out of the air bags using a syringe to measure the CH_4_ concentration according to the gas chromatographic method.

### 2.4. Sampling

After 6, 12, 24, 48, and 72 h of incubation in the manual system, the contents of each bottle were filtered through a nylon bag (8 × 12 cm; 42 μm pore size) and dried at 105 °C to determine the in vitro dry matter disappearance (IVDMD). Then, the culture fluids (6 × 1.0 mL) were sampled into DNase-free polypropylene tubes and stored at −80 °C for later analysis of VFA, nitrocompounds, microbial populations, *mcr*A (methyl coenzyme-M reductase subunit A) gene expression, coenzyme F_420_ content, and coenzyme F_430_ content.

### 2.5. Measurement of VFA, Coenzyme, and Nitrocompound Contents

The culture fluids (1.0 mL) from each of the 5 aforementioned time stamps were mixed with 300 μL metaphosphoric acid solution (25%, w/v) for 30 min and centrifuged at 10,000× *g* for 15 min at 4 °C. Supernatants (0.5 mL) were injected into gas chromatography to determine the concentrations of acetate, propionate, isobutyrate, butyrate, isovalerate, and valerate [11]. 

Following the description of Reuter et al. [13], coenzyme F_420_ was determined and expressed as fluorescence intensity. Assays were performed at 37 °C anaerobically in the dark. Culture fluid samples (1.0 mL) were stirred continuously and boiled at 95 °C in water bath for 30 min. Fluid aliquots were then centrifuged at 10,000× *g* for 10 min, and a volume of 500 μL from supernatants was mixed with 1 mL of isopropanol. Subsequently, the mixture was precipitated for 2 h and centrifuged at 10,000× *g* again for 15 min. Finally, the fluorescence intensity of the supernatants was measured at 420 nm by the fluorescence spectrophotometer (Thermo Fisher Scientific Co., Ltd., shanghai, China). Coenzyme F_430_ content was examined via the ultraviolet/visible spectrum by determining the loss of absorbance [14]. Briefly, the culture fluids were quenched with equal volumes of methanol and centrifuged aerobically at 6153× *g* for 20 min in dim light. The precipitate was discarded and the supernatants were determined colorimetrically using a spectrophotometer at 430 nm (Laspec Technology Co., Ltd., shanghai, China). Coenzyme F_430_ content was expressed as the relative absorbance of coenzyme F_430_ at 430 nm.

The contents of NE, NEOH, and NPOH were determined colorimetrically by a spectrophotometer (Laspec Technology Co., Ltd., shanghai, China) [15]. The culture fluids (1 mL) were firstly centrifuged at 10,000× *g* for 15 min. Supernatants (50 μL) were then diluted with 2 mL of distilled water and mixed with 100 μL of NaOH (0.65 M) and 100 μL of diazotized p-nitroaniline. Finally, the absorbances of the culture fluids were measured at a wavelength of 405 nm.

### 2.6. Analysis of mcrA Gene Expression

Total RNA of culture fluid was extracted by RNeasy Mini kit (Tiangen Biotech, Beijing, China) using an RNase-Free DNase Set (Qiagen). The detailed procedures for analysis of *mcr*A gene expression were described by Guo et al. [16]. The specific primer sets for the *mcr*A gene (F: 5’-TTCG GTGG ATCD CARA GRGC-3’, R: 3’-GBAR GTCG WAWC CGTA GAAT CC-5’) and the 16S rRNA gene (F: 5’-CGGC AACG AGCG CAAC CC-3’, R: 3’-CCAT TGTA GCAC GTGT GT AG CC-5’) were applied as described by Denman et al. [17] and Denman and McSweeney [18]. The 2^-∆∆Ct^ method was used for the expression analysis of the *mcr*A gene, with the 16S rRNA set as the reference gene.

### 2.7. Microbial Population Analysis with Real-Time PCR

Total DNA of culture fluid (1 mL) was extracted with the FastDNA kit and FastPrep instrument (Tiangen® Biotech, Beijing, China) by a bead-beating method as described by Denman and McSweeney [18]. According to the real-time PCR method [18], enumeration of total bacteria, total methanogens, *Methanobacteriales*, *Methanococcales*, *Methanomicrobiales*, protozoa, fungi, *Ruminococcus flavefaciens*, *Ruminococcus albus,* and *Fibrobacter succinogenes* was measured using the Bio-Rad Multicolor Real-Time PCR Detection System (Bio-rad Company, CA, USA) with qReal Master Mix SYBR® Green (Tiangen® Biotech, Beijing, China). The primer sets for the detection and enumeration of the microbial populations were described by Denman and McSweeney [18], Zhou et al. [19], and Yu et al. [20]. The abundance of the microbial population was expressed as a proportion of total estimated rumen bacterial (16S rDNA) according to the following equation: relative quantification = 2 ^−(CT target - CT total bacteria)^, where CT represents the threshold cycle. 

### 2.8. Data Calculation and Statistical Analysis

Cumulative gas production data from the AGRS were fitted according to an exponential model as described by France [21]. In addition, the average gas production rate (AGPR) (mL/h) and hydrogen recovery (2Hrec) were also calculated following the description by Zhang et al. [22].

The data for gas production, IVDMD, fermentation gas composition, coenzyme contents, and VFAs were subjected to analysis of variance with the MIXED model procedure of SAS (Statistical Analysis for Windows, SAS Institute Inc., Cary, NC, USA). The model was applied as *Y_ijk_* = *μ* + *R_i_* + *N_j_* + *T_k_* + (*N* × *T*) *_jk_* + *e_ijk_*,, where *Y_ijk_* is the dependent variable, μ represents the overall mean, *R_i_* is the effect of the experimental run, *N_j_* is the effect of the nitrocompound treatment, *T_k_* is the effect of the incubation time, *N × T* are the interactions between the nitrocompounds and the incubation time, and *e_ijk_* is the residual. The data for microbial abundance analysis were applied according to the model *Y_ijk_* = *μ* + *R_i_ + N_j_* + *e_ijk_*, where *Y_ijk_* is the dependent variable, μ represents the overall mean, *R_i_* is the effect of the experimental run, *N_j_* is the effect of the nitrocompound treatment, and *e_ijk_* is the residual. Least square means and standard error (SEM) were calculated, and treatment differences were estimated using a multiple comparisons test (Tukey/Kramer). Correlation analyses between variables were performed using the CORR procedure of SAS. Significance was declared at *p* < 0.05.

## 3. Results

### 3.1. IVDMD and Gas Production Kinetics

As the incubation time increased, both IVDMD (Figure 1a) and gas production (Figure 1b) continuously increased. Addition of NE, NEOH, and NPOH slowed down the fermentation process and caused IVDMD to decline (Figure 1a, *p* < 0.01). 

Regarding the kinetics of gas production, NE, NEOH, and NPOH addition decreased asymptotic gas production (A) (Table 1, *p* < 0.01), while NE and NEOH addition increased the fractional gas production rate (c, h^−1^). Neither NE nor NEOH addition altered AGPR, but NPOH decreased AGPR compared to the control.

### 3.2. Fermentation Gas Composition

**Figure 2:**
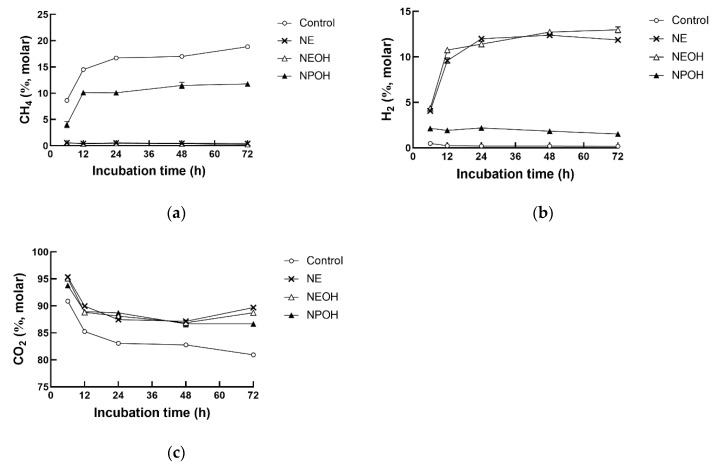
Effect of NE, NEOH, and NPOH addition to culture fluids on CH_4_ (**a**), H_2_ (**b**), and CO_2_ (**c**) production of grain-rich feed incubated with rumen fluids obtained from lactating dairy cows. NE: nitroethane; NEOH: 2-nitroalcohol; NPOH: 2-nitro-1-propanol. Statistical analyses showed the effects of nitrocompounds on CH_4_, H_2_, and CO_2_*,* with correlations significant at *p* < 0.01.

H_2_ accumulation in the NE, NEOH, and NPOH groups was far greater than in the control group (Table 1, *p* < 0.01). The molar proportions of CO_2_ were increased (5.7%, 5.2%, and 5.4%) by NE, NEOH, and NPOH compared to the control, whereas the molar proportions of CH_4_ were notably decreased (96.7%, 96.7%, and 41.7%, Table 1). An interaction between the nitrocompound treatment and the incubation time was observed for gas composition (*p* < 0.01). The molar proportion of CH_4_ constantly increased with increasing incubation time in the control and NPOH groups, but it was at a pretty low level in both the NE and NEOH groups (*p* < 0.01, Figure 2a). In contrast, molar H_2_ production in the NE and NEOH groups continuously increased with increasing incubation time and was always far greater than the control and NPOH throughout the incubation (*p* < 0.01, Figure 2b). In addition, as the incubation time increased, the molar proportion of CO_2_ gradually decreased in all groups, and it was greater in the nitrocompound-treated cultures than the control (*p* < 0.01, Figure 2c).

### 3.3. Coenzymes Related to CH_4_ Production

Compared to the control, both coenzyme F_420_ fluorescence intensity and F_430_ ultraviolet (UV) absorbance declined in the NE, NEOH, and NPOH groups (Table 2, *p* < 0.01). In addition, NE, NEOH, and NPOH addition decreased *mcr*A gene expression by 83.1%, 79.7%, and 53.5%, respectively. 

**Figure 3:**
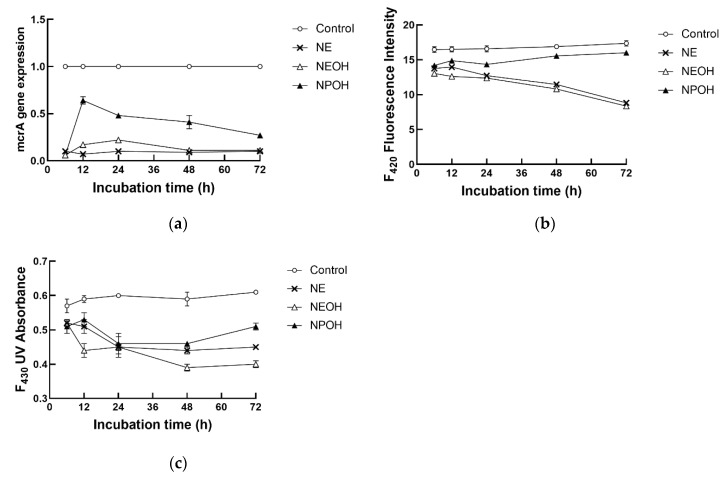
Effect of NE, NEOH, and NPOH addition to culture fluids on *mcr*A gene expression (**a**) and the contents of coenzyme F_420_ (**b**) and coenzyme F_430_ (**c**) of grain-rich feed incubated with rumen fluids obtained from lactating dairy cows. NE: nitroethane; NEOH: 2-nitroalcohol; NPOH: 2-nitro-1-propanol. Statistical analyses showed that the effects of nitrocompounds were significant at *p* < 0.01.

An interaction between the nitrocompound treatment and the incubation time was evident for *mcr*A gene expression and coenzyme F_420_ and F_430_ contents (*p* < 0.01). As the incubation time increased, *mcr*A gene expression in the NPOH group relative to the control peaked at 12 h during the 72 h incubation, and it was continuously far lower in the NE and NEOH groups than in the control and NPOH groups (Figure 3a). Coenzyme F_420_ fluorescence intensity gradually declined in both the NE and NEOH groups with increasing incubation time, and it was continuously lower in the nitrocompound-treated cultures than the control (Figure 3b). The F_430_ ultraviolet (UV) absorbance continuously decreased in the NE and NEOH groups with increasing incubation time (Figure 3c), but it fluctuated during 72 h incubation in the NPOH group. In addition, the coenzyme F_430_ content was constantly lower in the nitrocompound-treated cultures than the control. 

### 3.4. Fermentation Characteristics

**Figure 4:**
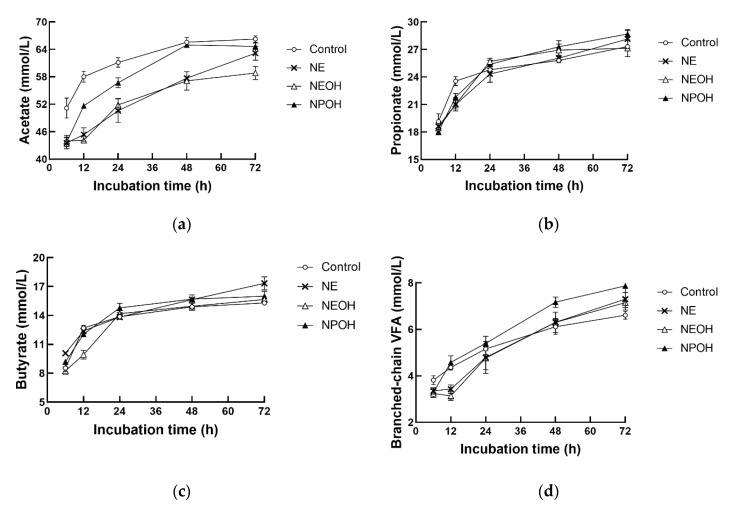
Effect of NE, NEOH, and NPOH addition to culture fluids on acetate (**a**), propionate (**b**), butyrate (**c**), and branched-chain VFA (**d**) of grain-rich feed incubated with rumen fluids obtained from lactating dairy cows. NE: nitroethane; NEOH: 2-nitroalcohol; NPOH: 2-nitro-1-propanol. Statistical analyses showed that the effects of nitrocompounds on acetate and butyrate were significant at *p* < 0.01, but the effects of nitrocompounds on propionate and branched-chain VFAs were not significant.

Concentrations of total VFAs and acetate were lower in the NE, NEOH and NPOH groups than in the control (Table 2, *p* < 0.01), whereas the concentration of butyrate increased with NE addition (Table 2, *p* < 0.01). NE, NEOH, and NPOH addition had no significant influence on propionate and branched-chain VFAs (BCVFAs). Compared to the control, NE, NEOH, and NPOH addition decreased 2Hrec by 30.2%, 28.3%, and 12.3%, respectively (Table 2, *p* < 0.01). An interaction between the nitrocompound treatment and the incubation time was observed for total VFA, acetate, propionate, and butyrate. The concentrations of total VFAs, acetate, propionate, butyrate, and BCVFA were continuously increased in all groups with increasing incubation time (Figure 4). In addition, concentrations of acetate were continuously lower in nitrocompound-treated cultures than in the control, whereas the concentrations of propionate, butyrate, and BCVFAs fluctuated during 72 h incubation.

### 3.5. Microbial Populations

Real-time PCR results showed that NE, NEOH, and NPOH addition decreased the relative populations of total methanogens, *Methanobacteriales*, *Methanomicrobiales,* and *Fibrobacter succinogenes* (Table 3, *p* < 0.05). Compared to the control, the relative populations of total methanogens decreased by 49.2%, 36.9%, and 41.5% in the NE, NEOH, and NPOH groups, and the populations of *Methanobacteriales* decreased by 46.1%, 35.9%, and 17.9%, respectively. In addition, the relative populations of fungi tended to decrease according to nitrocompound treatment (*p* = 0.09). Compared with control, the populations of *R. albus* increased by 50.0% and 50.0% in the NEOH and NPOH groups, and the populations of *R. flavefaciens* decreased by 84.5% and 70.7% in the NE and NEOH groups. However, the populations of protozoa were not affected (*p* = 0.29).

### 3.6. Correlation between CH_4_ Production and mcrA Gene Expression/Coenzyme Contents

CH_4_ production was positively correlated with *mcr*A gene expression (Table 4; r = 0.88, *p* < 0.05), coenzyme F_420_ content (Table 4; r = 0.74, *p* < 0.01), and coenzyme F_430_ content (Table 4; r = 0.24, *p* < 0.05). In addition, there was a positive correlation between *mcr*A gene expression and the coenzyme F_420_ content (r = 0.78, *p* < 0.01), and between *mcr*A gene expression and the coenzyme F_430_ content (r = 0.22, *p* < 0.05). A positive correlation was also observed between the coenzyme F_420_ content and the coenzyme F_430_ content (r = 0.26, *p* < 0.05).

### 3.7. Disappearance of Nitrocompounds

**Figure 5:**
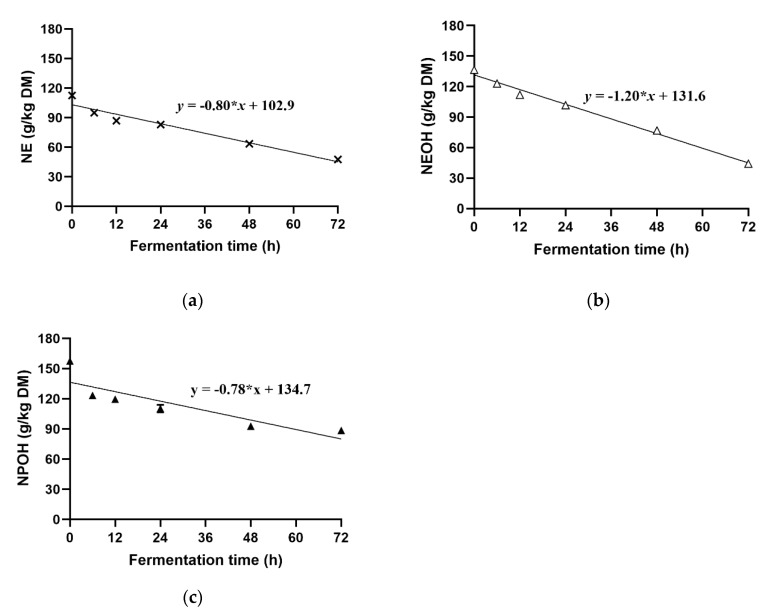
Variation of NE (**a**), NEOH (**b**), and NPOH (**c**) in culture fluids during 72 h incubation of grain-rich feed incubated in batch cultures of mixed rumen microorganisms. DM: dry matter; NE: nitroethane; NEOH: 2-nitroalcohol; NPOH: 2-nitro-1-propanol.

Concentrations of NE, NEOH, and NPOH decreased with increasing incubation time (Figure 5). The disappearance of nitrocompounds was fitted to a linear model, and the nitrocompound degradation rate was ranked as NEOH (−1.20) > NE (−0.80) > NPOH (−0.78).

## 4. Discussion

### 4.1. Effects of Nitrocompounds on IVDMD and Gas Production

A majority of CH_4_ inhibition strategies tend to compromise fermentative efficiency, resulting in the reduction of certain digestive processes [11,23]. In the present study, the addition of NE, NEOH, and NPOH caused notable reductions of IVDMD, gas production, and total VFAs, indicating that the activity of the microbes responsible for the degradation of substrates was inhibited by these nitrocompounds. The inclusive level of nitrocompounds was determined according to previous studies [11,24], however, a remarkable reduction in total VFA production occurred in the NE, NEOH, and NPOH groups, indicating that the fermentative bacterial population may be sensitive to a 10 mM dose level of nitrocompounds.

### 4.2. Effects of Nitrocompounds on CH_4_ Production

The antimethanogenic activity of NE, NEOH, and NPOH was previously observed. For instance, more than 90% of CH_4_ production was inhibited by NE, NEOH, and NPOH at a concentration of 9~24 mM after 24 h incubation [5,24]. The addition of NE, NEOH, and NPOH in the present study caused reductions in CH_4_ production of 96.7%, 97.2%, and 39.5%, respectively, which agreed with the results of earlier studies [24,25]. In addition, both NE and NEOH were more effective in reducing ruminal CH_4_ production than NPOH.

The nitro group is strongly electron-withdrawing. Because of this property, nitrocompounds may be able to serve as electron acceptors within ruminal microbes to reduce CH_4_ production. Early studies done by Anderson et al. [26] revealed that nitrocompounds could serve as electron acceptors within ruminal microbes to inhibit CH_4_ production. However, nitrocompounds also directly inhibit ruminal methanogenesis [27]. The first author in a review summarized the anti-methanogenic roles of nitrocompounds and their potential inhibitory action modes in terms of VFA production, hydrogen accumulation, formate oxidation and ferredoxin-linked hydrogenase activity [28]. Notable accumulation of H_2_ in NE-, NEOH-, and NPOH-treated cultures and CH_4_ production inhibition were observed in the present study. Similarly, Anderson et al. [25] also found that accumulations of H_2_ were higher in nitrocompound-supplemented cultures than in controls after 24 h of incubation. In the unperturbed rumen, H_2_ is usually present at approximately 1 μmol/L (0.1 kPa); however, it often increases to concentrations that inhibit hydrogenase activity (1 kPa), while ruminal CH_4_ production is inhibited due to declined H_2_ consumption by methanogens [23]. Considering that hydrogenases are reversible enzymes that can catalyze either the production or oxidation of H_2_, the authors in the present study speculated that nitrocompounds may have inhibited H_2_-oxidation hydrogenase activity as well, which was in accordance with Angermaier and Simon [29], who reported that NEOH inhibited ferredoxin-linked hydrogenase uptake activity. Likewise, Anderson et al. [30] also noted that NE, NEOH, NPOH, and NPA each inhibited oxidation of H_2_ or formate when these reductants were added in excess (60 mM) to mixed cultures of rumen microbial populations, thereby implicating a possible mechanism of activity against ruminal methanogenesis. 

Consistent with the different levels of CH_4_ inhibition efficiency, NE and NEOH were shown to be nearly equally effective in promoting H_2_ accumulation in vitro, with both of them promoting H_2_ accumulation more effectively than NPOH. The molar proportions of H_2_ in total fermentation gas production were 9.8%, 10.5%, and 2.0% in the NE, NEOH, and NPOH groups, respectively. However, the decreasing extent of 2Hrec in the present study was far less than that of CH_4_ production. The fate of the remaining H_2_ is not known with certainty, however, the consumption of reducing equivalents may occur during anabolic processes, including cell growth, intracellular polyhydroxyalkonoate, or extracellular polysaccharide production [25].

### 4.3. Effects of Nitrocompounds on VFA Production

In order to compensate for the disruption of electron flow to ruminal methanogenesis, some CH_4_ inhibitors, such as sodium sulfite, organic halides, and monensin, cause notable increases in propionate production during fermentation, which is frequently accompanied by decreased acetate [23]. However, NE, NEOH, and NPOH addition in the present study had little effect on propionate produced by ruminal populations. Therefore, the results indicate that the reducing equivalents produced during ruminal fermentation were not necessarily directed toward increased production of propionate. In addition, the concentrations of acetate in the present study were decreased in the nitrocompound-treated cultures, along with the reduction of CH_4_ production. It is well recognized that acetate accompanies H_2_ production, and the latter can be used for CH_4_ formation by methanogenic archaea, with CH_4_ being positively associated with the acetate to propionate ratio [31].

### 4.4. Effects of Nitrocompounds on Methane Production and Related Changes in Microbial Populations and Coenzyme Contents

Methanogens are a unique group of ruminal microbes that generate CH_4_ as a stoichiometric end-product of their metabolism. Methanogen populations are generally closely associated with CH_4_ production [32]. In the present study, NE, NEOH, and NPOH addition decreased total methanogens by 49.2%, 36.9%, and 41.5%, respectively, and the authors speculated that NE, NEOH, and NPOH likely exerted a direct inhibition of ruminal methanogenesis via direct suppression of methanogens. *Methanobacteriales* are the predominant populations and constitute the major portion of methanogen community in ruminants, being the second most prevalent archaea in the rumen ecology [33]. Presently, both the relative abundances of *Methanobacteriaceae* and *Methanomicrobiales* were reduced with NE, NEOH, and NPOH addition, but different sensitivity responses to these nitrocompounds were observed. *Methanobacteriaceae* reduction varied in the order of NE (−46.2%) > NEOH (−35.9%) > NPOH (−17.9%), whereas *Methanomicrobiales* reduction varied in the order of NE (−67.6%) > NPOH (−64.1%) > NEOH (−33.1%). 

Rumen protozoa constitute only a small portion of ruminal microorganisms, however, they play important roles in feed degradation and making energy and protein available to the hosts. The RT-PCR results showed no apparent decrease in the protozoal population in the NE-, NEOH- and NPOH-treated cultures, indicating that there were no adverse effects of the nitrocompounds on the rumen protozoa populations.

Few studies have determined microbial responses to nitrocompounds. Anderson et al. [26] noted that 3-NPOH and NPA modestly inhibited total culturable anaerobes from bovine rumens, but inhibited microbes were not characterized. Rumens harbor different types of bacteria, which are most actively involved in plant fiber degradation. It is well recognized that the depression of cellulolysis can decrease the rate and extent of neutral detergent fiber digestion [34], and the inhibition to cellulolytic microorganisms (*R. flavefaciens*, *F. succinogenes*) by nitrocompounds may explain why decreased IVDMD was observed in the cultures supplemented with NE, NEOH, or NPOH. Fibrolytic bacteria families, including *R. flavefaciens*, *R. albus*, and *F. succinogenes,* have vbeen shown to release many fibrolytic enzymes and promote H_2_, acetate and formic acid production for methanogen utilization [35]. Real-time PCR results showed that the relative populations of the methanogens *R. Flavefaciens* and *F. succinogenes* decreased with nitrocompound addition, suggesting that the mutual-aid interaction between methanogens and fibrolytic bacteria might be one reason why *R. Flavefaciens* and *F. succinogenes* decreased along with methanogen inhibition by NE, NEOH, and NPOH. The rumen protozoa produce fermentation end-products similar to those made by the bacteria, particularly acetate, butyrate, and H_2_. They utilize large amounts of starch at one time and can store it in their bodies. The corn-rich substrate applied in the present study may have been adequate to maintain the growth of protozoa, which may explain why the nitrocompounds had no negative effect on the abundance of protozoa. In the present study, the abundance of *R. albus* increased with NEOH and NPOH addition, while anaerobic rumen fungi decreased with nitrocompound supplementation. This phenomenon could be attributed to the antagonistic association between ruminal fungi and cellulolytic bacteria [36]. In summary, although nitrocompounds could change the relative abundance of some microbial populations, the differences in diversity and metabolic activity in response to NE, NEOH, and NPOH need further investigation in order to determine the maximal inhibitory effect on CH_4_ production with minimal adverse effects on rumen fermentation.

The hydrogenotrophic, methylotrophic, and acetoclastic pathways are the three major pathways for ruminal CH_4_ production. The biochemical reactions and enzyme profiles involved in methanogenesis are well identified and described [37]. Methyl-coenzyme M reductase (*mcr*) is a key enzyme responsible for catalyzing the CH_4_-producing step in the process of methanogenesis. As a gene encoding the alpha-subunit of *mcr*, *mcr*A was evolutionarily highly conserved, probably due to functional constraints on the catalytic activity of *mcr* [35]. Recently, the determination of *mcr*A gene expression was accepted for *mcr* activity measurement [16]. A positive correlation between decreased *mcr*A gene expression and decreased molar CH_4_ proportions was found in the present study, suggesting that NE, NEOH, and NPOH inhibited CH_4_ production by decreasing *mcr* activity. In addition, the activity of *mcr* is dependent mainly on the unique nickel-containing tetrapyrrole known as coenzyme F_430_ [38]. As reported by Gunsalus and Wolfe [39], coenzyme F_430_ is a yellow nonfluorescent compound released from *mcr*, with an absorption maximum at 430 nm on its UV-Vis absorption spectrum. In the present study, NE, NEOH, and NPOH addition also reduced the content of coenzyme F_430_. Coenzyme F_420_ is of importance for methanogenesis and can act as an indicator for methanogenic activity [8,40]. The coenzyme F_420_ content in this study also decreased in the nitrocompound-treated cultures and was accompanied by CH_4_ reduction, with the anti-methanogenic activity ranked as NEOH > NE > NPOH. A possible reason for these results might be due to the toxic action of nitrocompounds to methanogens [24,27]; this needs further clarification. 

### 4.5. Degradation of NE, NEOH, and NPOH

Decreased concentrations of NE, NEOH, and NPOH were observed with increasing incubation time in all of the nitrocompound-treated cultures, thus confirming the presence of competent nitrocompound-degrading microbes within the incubations. A previous study by Anderson et al. [26] revealed that most of the rumen microorganisms tolerated nonlethal concentrations of naturally occurring nitrocompounds. In addition, the degradation rate of nitrocompounds can be enhanced via exposure to nitropropionic acid-containing forages. This phenomenon could be ascribed the improvement of nitro-degrading activity or enrichment in numbers of nitro-degrading microorganisms. Until now, *Denitrobacterium detoxificans* was recognized as a unique nitrocompound-reducing microbe which oxidizes reducing substrates, including H_2_, formate, and lactate, to reduce nitrocompounds to their respective amines and minor nitrites [41]. Consequently, *D. detoxificans* has the potential to outcompete ruminal methanogens for available reductants [15]. In the present study, the nitrocompound disappearance rate was ranked as NEOH (−1.20) > NE (−0.80) > NPOH (−0.78), suggesting that the rumen microbes presented divergent metabolic capabilities regarding nitrocompound degradation, thus partially explaining why NE, NEOH, and NPOH showed different competition for methanogenesis-produced reductants.

## 5. Conclusions

Along with a dramatic increase in H_2_ accumulation, both NE and NEOH were shown to be more effectivene in inhibiting methanogenesis than NPOH. Although nitrocompound addition decreased acetate and total VFA production, it had no negative effect on propionate. In addition, NE, NEOH, and NPOH addition decreased the population abundance of total methanogens, *Methanobacteriales*, and *Methanomicrobiales*, also causing decreases in *mcr*A gene expression and coenzyme F_420_ and F_430_ contents. The results provided evidence that NE, NEOH, and NPOH could reduce methanogen populations and dramatically decrease *mcr*A gene expression and coenzyme F_420_ and F_430_ contents with different magnitudes to reduce overall ruminal CH_4_ production. 

## Figures and Tables

**Table 1:** Effect of NE, NEOH, and NPOH addition (10 mmol/L) to culture fluids on gas production kinetics and fermentation gas composition during 72 h incubation.

Items ^1^	Treatment ^2^				SEM	*p* ^3^		
	Control	NE	NEOH	NPOH		Treatment	Time	Trt × Time
Gas production kinetics								
*A*, mL/g DM	199 ^a^	178 ^b^	170 ^c^	173 ^bc^	1.96	<0.01	-	-
*c*, h^−1^	0.09 ^b^	0.11^a^	0.12 ^a^	0.10 ^b^	0.02	<0.01	-	-
*T*_1/2_, h	3.1	3.1	3.0	3.1	0.02	0.07	-	-
AGPR, mL/h	13.3 ^a^	13.9 ^a^	13.9 ^a^	11.9 ^b^	0.29	<0.01	-	-
Fermentation gas composition, %								
CH_4_	15.1 ^a^	0.5 ^c^	0.5 ^c^	8.8 ^b^	0.39	<0.01	<0.01	<0.01
H_2_	0.3 ^d^	9.8 ^b^	10.5 ^a^	2.0 ^c^	0.23	<0.01	<0.01	<0.01
CO_2_	84.6 ^b^	89.4 ^a^	89.0 ^a^	89.2 ^a^	0.36	<0.01	<0.01	0.01

^a–d^ Means with different superscripts within a row are significantly different (*p* < 0.05); ^1^ *A*: asymptotic gas production; *c*: fractional gas production rate; T_1/2_: the time when half of A occurred; AGPR: average gas production rate. ^2^ NE: nitroethane; NEOH: 2-nitroalcohol; NPOH: 2-nitro-1-propanol. ^3^ Interaction effect between treatment and incubation time.

**Table 2:** Effect of NE, NEOH, and NPOH addition (10 mmol/L) on coenzyme content, *mcr*A gene expression, and volatile fatty acid (VFA) production in fermentation fluids across different incubation times (6, 12, 24, 48, and 72 h).

Items ^1^	Treatment ^2^				SEM	*p* ^3^		
	Control	NE	NEOH	NPOH		Treatment	Time	Trt × Time
*Coenzyme content*								
F_420_	16.7 ^a^	11.9 ^c^	11.4 ^c^	15.2 ^b^	0.23	<0.01	<0.01	<0.01
F_430_	0.59 ^a^	0.48 ^b^	0.44 ^c^	0.50 ^b^	0.02	<0.01	<0.01	0.01
*mcr*A expression	1 ^a^	0.11 ^c^	0.12 ^c^	0.42 ^b^	0.02	<0.01	<0.01	<0.01
Total VFA, mmol/L	102.8 ^a^	93.7 ^c^	94.7 ^bc^	97.4 ^b^	1.64	<0.01	<0.01	<0.01
Acetate, mmol/L	60.9 ^a^	52.1 ^c^	53.1 ^c^	54.7 ^b^	1.31	<0.01	<0.01	0.02
Propionate, mmol/L	24.0	23.1	24.2	23.9	0.53	0.33	<0.01	0.02
Butyrate, mmol/L	12.8 ^bc^	13.5 ^a^	12.5 ^c^	13.2 ^ab^	0.30	<0.01	<0.01	<0.01
BCVFA, mmol/L	5.1	5.0	4.9	5.6	0.31	0.14	<0.01	0.14
2Hrec, %	67.6 ^a^	47.2 ^c^	48.5 ^c^	59.3 ^b^	0.87	<0.01	<0.01	<0.01

^a–d^ Means with different superscripts within a row are significantly different (*p* < 0.05); ^1^ F_420_: F_420_ fluorescence intensity; F_430_: UV absorbance of coenzyme F_430_; BCVFA: branch-chained volatile fatty acids; 2Hrec: hydrogen recovery. ^2^ NE: nitroethane, NEOH: 2-nitroalcohol; NPOH: 2-nitro-1-propanol. ^3^ Interaction effect between treatment and incubation time.

**Table 3:** Effects of NE, NEOH, and NPOH addition (10 mmol/L) to culture fluids on microorganism relative populations after 72 h incubation.

Items	Treatment					
	Control	NE	NEOH	NPOH	SEM	*p*
Total methanogen	0.65 ^a^	0.33 ^b^	0.41 ^b^	0.38 ^b^	0.041	0.04
*Methanobacteriales*	0.39 ^a^	0.21 ^c^	0.25 ^bc^	0.32 ^b^	0.013	0.02
*Methanomicrobiales*, ×10^−3^	14.2 ^a^	4.6 ^c^	9.5 ^b^	5.1 ^c^	0.985	0.01
*Methanococcales*, ×10^−3^	0.44 ^bc^	0.56 ^ab^	0.65 ^a^	0.38 ^c^	0.041	0.03
Fungus, ×10^−3^	1.82 ^a^	0.37 ^b^	1.25 ^ab^	1.14 ^ab^	0.240	0.09
Protozoa, ×10^−3^	0.28	0.29	0.43	0.22	0.068	0.29
*Ruminococcus albus*	0.12 ^b^	0.10 ^b^	0.18 ^a^	0.18 ^a^	0.012	<0.01
*Ruminococcus flavefaciens*	0.58 ^a^	0.09 ^b^	0.17 ^b^	0.43 ^a^	0.056	0.01
*Fibrobacter succinogenes*, ×10^−3^	3.33 ^a^	1.39 ^b^	0.97 ^b^	0.56 ^b^	0.232	<0.01

^a–c^ Means with different superscripts within a row are significantly different (*p* < 0.05). NE: nitroethane; NEOH: 2-nitroalcohol; NPOH: 2-nitro-1-propanol.

**Table 4:** Correlations between CH_4_ production, *mcr*A gene expression, coenzyme F_420_ content, and coenzyme F_430_ content, regardless of the type of nitrocompound added.

Items	*mcrA*	F_420_	F_430_
methane production	0.88 *	0.74 **	0.24 *
*mcrA*		0.78 **	0.22 *
F_420_			0.26 *

Significance: * *p* <0.05, ** *p* <0.01.
